# A case report of multiple cerebral abscess formation complicating serogroup B Neisseria meningitidis meningitis

**DOI:** 10.1186/s12879-019-4509-y

**Published:** 2019-10-21

**Authors:** Ciara O’Connor, Aedin Collins, Eilish Twomey, Conor Hensey, John Caird, Patrick J. Gavin

**Affiliations:** 1 Department of Clinical Microbiology, Temple Street Children’s University Hospital, Dublin 1, Ireland; 2 Department of Infectious Diseases, Temple Street Children’s University Hospital, Dublin 1, Ireland; 3 Department of Radiology, Temple Street Children’s University Hospital, Dublin 1, Ireland; 4 Deparment of General Paediatrics, Temple Street Children’s University Hospital, Dublin 1, Ireland; 5 Department of Neurosurgery, Temple Street Children’s University Hospital, Dublin 1, Ireland

**Keywords:** Meningitis, Cerebral abscess, *Neisseria meningitidis*, Serogroup B, Meningococcus, Seizures

## Abstract

**Background:**

Invasive meningococcal disease (IMD) presenting with meningitis causes significant mortality and morbidity. Suppurative complications of serogroup B meningococcal sepsis are rare and necessitate urgent multidisciplinary management to mitigate long-term morbidity or mortality.

**Case presentation:**

We present a rare case of invasive meningococcal disease in a 28-month old boy complicated by multiple abscess formation within a pre-existing antenatal left middle cerebral artery territory infarct. Past history was also notable for cerebral palsy with right hemiplegia, global developmental delay and West syndrome (infantile spasms). Two craniotomies were performed to achieve source control and prolonged antimicrobial therapy was necessary. The patient was successfully discharged following extensive multidisciplinary rehabilitation.

**Conclusions:**

Longstanding areas of encephalomalacia in the left MCA distribution may have facilitated the development of multiple meningococcal serogroup B abscess cavities in the posterior left frontal, left parietal and left temporal lobes following an initial period of cerebritis and meningitis. A combination of chronic cerebral hypoperfusion and some degree of pre-existing necrosis in these areas, may also have facilitated growth of *Neisseria meningitidis,* leading ultimately to extensive cerebral abscess formation following haematogenous seeding during meningococcemia. In this case report we review similar cases of cerebral abscess or subdural empyema complicating serogroup B meningococcal meningitis.

## Background

*Neisseria meningitidis* is an opportunist Gram-negative pathogen and commensal of the nasopharynx and upper respiratory tract. Transmission is via aerosol, droplets or direct contact with infected respiratory secretions. The prevalence of asymptomatic carriage of all serotypes of *N. meningitidis* increases with age, from 5% in childhood to 20% in late teenage years [1]. There are 13 antigenically distinct serogroups of meningococci and most disease-associated strains belong to serogroups A, B, C, X, Y or W135. The incidence of meningococcal disease varies worldwide but is estimated at 0.3–3 cases per 100,000 in Europe and North America, with a predominance of serogroups B and C. Overall, the incidence of meningococcal disease continues to decline in countries where vaccines targeting serogroups C and more recently B, have been introduced [2].

Invasive meningococcal disease (IMD) presenting with meningitis causes significant mortality, with a 10% case fatality rate in the very young, and morbidity in up to 20% of survivors, predominantly neurological sequelae (seizures, cognitive impairment), visual and/or auditory impairment and amputation of necrotic digits and/or limbs [3]. By contrast, acute suppurative complications of *N. meningitidis* meningitis occur in less than 1% of cases and are rarely reported [4, 5]. We report the successful treatment of multiple cerebral abscesses associated with serogroup B meningococcal meningitis in a young infant with a prior cerebral infarct and complex medical history. The overall aims of presenting this case report are to increase awareness of the increased risk of cerebral complications of meningococcal sepsis in patients with previous intracerebral neurovascular events and to demonstrate the role of advances in molecular diagnostics, permitting PCR detection of *N. meningitidis* DNA despite preceding antimicrobial therapy.

## Case presentation

A 28-month old boy presented with a 24-h history of lethargy, pyrexia, mottled peripheries and decreased responsiveness. Past history was notable for antenatal left middle cerebral artery (MCA) infarct, cerebral palsy with right hemiplegia, global developmental delay and West syndrome (infantile spasms). He was up-to-date with vaccinations. Epilepsy was poorly controlled with up to 30 seizures per day despite maintenance clobazam, lacosamide, sodium valproate and levetiracetam treatment.

**Fig. 1:**
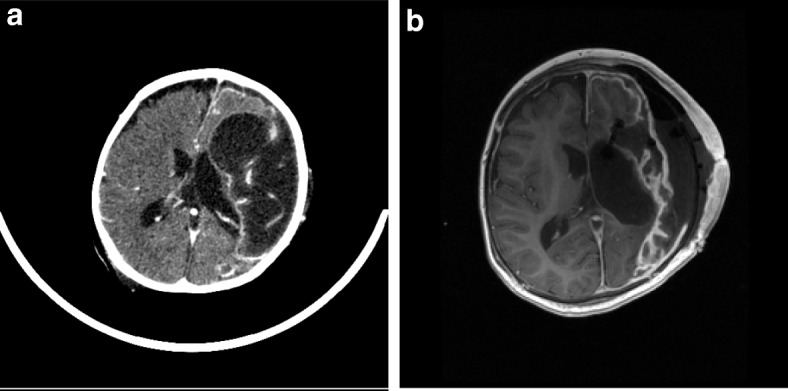
**a**: Axial contrast-enhanced CT day 10 of illness reveals an extensive area of cystic encephalomalacia in the left middle cerebral artery territory with marginal enhancement. Pial enhancement is evident of the entire left cerebral hemisphere. There is longstanding ex vacuo dilatation of the left lateral ventricle and new mass effect with shift of the anterior falx to the right. **b**: Axial contrast-enhanced T1 MRI on day 21 after two neurosurgical interventions. The extensive left craniotomy, evacuation and debridement of the infected encephalomalacic tissue is evident with pronounced pial and leptomeningeal enhancement of the left cerebral hemisphere. There is discontinuity of the dura with overlying extradural fluid and air. An extra-ventricular drain tip is located in the anterior left lateral ventricle

On examination he had neck stiffness and was haemodynamically unstable with persistent tachycardia and hypotension. He was coagulopathic (platelets 94 × 10 [6]/L, INR 2.5, fibrinogen 4.03 g/L, prothrombin time 16.8 s), with elevated white cell count (WCC) 28.9 × 10 [6]/L, neutrophilia (25.8 × 10 [6]/L), normal lymphocyte count (8.5 × 10 [6]/L) and c-reactive protein (CRP) 276 mg/L. He was transferred to the intensive care unit (day 0) where he required intubation, ventilation, fluid resuscitation and inotropic support. He was treated with intravenous (IV) ceftriaxone, vancomycin and aciclovir. Polymerase chain reaction (PCR) identified human metapneumovirus, parainfluenza type-3 and adenovirus in a nasal swab. Lumbar puncture demonstrated a macroscopic yellow appearance: WCC 142 (predominantly polymorphonuclear); glucose, 1.6 mmol/L; and protein, 5.46 g/L. No organisms were seen on Gram stain. Blood and cerebrospinal fluid (CSF) were culture negative (taken on antimicrobials) but PCR positive for *N. meningitidis* serogroup B. Antimicrobial treatment was rationalised to cefotaxime monotherapy. He subsequently became more encephalopathic with persistent pyrexia associated with rising blood WCC and CRP. Brain computed tomogram (CT) and magnetic resonance imaging (MRI) demonstrated multiple abscesses in the left MCA territory, cystic encephalomalacia of posterior left frontal, left parietal and left temporal lobes, and left hemispheric pial enhancement (Fig. 1a). Empiric treatment for cerebral abscess with IV meropenem, vancomycin and fluconazole was commenced.

A frameless stereotactic-guided endoscopic fenestration, drainage and washout of the cerebral abscesses was performed and a left external ventricular drain was sited (day 10). Further deterioration in neurological status necessitated an extensive fronto-temporal-parietal craniotomy with evacuation and debridement of residual collections and infected encephalomalacial tissue (day 16). Intraoperative fluid and parenchymal tissue was culture negative but PCR positive for *N. meningitidis* serogroup B and antimicrobial therapy was rationalised to meropenem monotherapy. A second craniotomy was performed for evacuation of a left frontal lobe abscess in the context of persistent swinging pyrexia (day 18). Intravenous linezolid was added for the treatment of a surgical site infection in the left parietal region, associated with a toxic shock-like syndrome. Intra-operative specimens were culture negative. The patient returned to theatre for repeat burrhole drainage of an intracerebral collection (day 20; Fig. 1b demonstrates clinical status post-operatively day 21). Intraoperative specimens were culture-negative again and were not sent for PCR analysis. He was discharged from ICU for extensive multidisciplinary rehabilitation (day 23). The patient was discharged home on day 40 to complete a further 6-weeks IV ceftriaxone. On outpatient follow-up, he is back to his baseline functioning status with no evidence of new neurological or auditory sequelae. Unfortunately, his seizures, which had disappeared in the immediate post-operative period, have since returned to their usual frequency.

## Discussion & Conclusions

The present case is an example of a rare meningococcal complication with a positive outcome. Following the introduction of *N. meningitidis* serogroup C conjugate (MenC) vaccine in 2000, meningococcal B disease has been the predominant cause of IMD in Ireland [7]. In 2015, the age specific incidence rate (ASIR) for meningococcal B disease in children aged between 1 and 4 years was 3.87/100,000 with a case fatality rate of less than 5% [8]. Our patient was non-immune as vaccination against meningococcal serogroup B would not have been offered to his age-cohort at the time of his presentation.

His complex pre-admission intracerebral neuropathology likely increased the risk of meningococcal abscess formation. We hypothesise that areas of encephalomalacia in the left MCA distribution (Fig. 1a, b) may have facilitated the development of multiple meningococcal serogroup B abscess cavities in the posterior left frontal, left parietal and left temporal lobes following an initial period of cerebritis and meningitis. A combination of chronic cerebral hypoperfusion and some degree of pre-existing necrosis in these areas, may also have facilitated growth of *N. meningitidis,* leading ultimately to extensive cerebral abscess formation following haematogenous seeding during meningococcemia. West syndrome is not known to confer an increased risk of meningitis [9].

Similar cases of cerebral abscess or subdural empyema complicating serogroup B meningococcal meningitis are rare (Additional file 1). Rothbaum et al. reported successful antimicrobial treatment and surgical drainage of cerebral abscess in an area of left temporal lobe hypoperfusion in a previously healthy 5-month old with serogroup B meningococcal septic shock [5]. And more recently, multiple cerebral abscesses were reported in association with serogroup B meningococcal meningitis in a five-day old neonate in India [6].

With regard to learning outcomes from the present case, we remain vigilant to the increased risk of cerebral complications of meningococcal sepsis in all children, but particularly those with previous intracerebral neurovascular events, and note again that diagnosis was facilitated by advances in molecular microbiology permitting PCR detection of *N. meningitidis* DNA despite 16 days of preceding antimicrobial therapy [8]. A previous study from this centre, reviewing 266 cases of adult and paediatric IMD over 8 years (2001-2008), demonstrated that 63% of cases of meningococcal blood stream infection and 69% of cases of meningitis were diagnosed by PCR alone, emphasising the necessity for clinicians to utilise rapid PCR testing where IMD is suspected, to reduce mortality and morbidity [10]. We also continue to promote meningococcal vaccination as a public health initiative.

## Additional file

**Additional file 1: Table S1.** Previous published papers describing meningococcal abscess formation

## Data Availability

No additional data are available.

## Abbreviations

CRP: C-reactive protein
CSF: Cerebrospinal fluid
CT: Computed tomogram
IMD: Invasive meningococcal disease
IV: Intravenous
MCA: Middle cerebral artery
MRI: Magnetic resonance imaging
PCR: Polymerase chain reaction
WCC: White cell count
