# Supplementary material for: A case report of multiple cerebral abscess formation complicating serogroup B Neisseria meningitidis meningitis
Source: BMC Infect Dis. 2019 Oct 21;19:863. doi: 10.1186/s12879-019-4509-y (PMC6802112; doi:10.1186/s12879-019-4509-y)
Supplement: Supplementary file 1 — Additional file 1: Table S1. Previous published papers describing meningococcal abscess formation [file 12879_2019_4509_MOESM1_ESM.docx]

Previous published papers describing meningococcal abscess formation

| Paper | DOI and/or Pubmed ID | Accessed | Not accessed | Published language |
| --- | --- | --- | --- | --- |
| [Chugh K](https://www.ncbi.nlm.nih.gov/pubmed/?term=Chugh%20K%5BAuthor%5D&cauthor=true&cauthor_uid=3278292), [Bhalla CK](https://www.ncbi.nlm.nih.gov/pubmed/?term=Bhalla%20CK%5BAuthor%5D&cauthor=true&cauthor_uid=3278292), [Joshi KK](https://www.ncbi.nlm.nih.gov/pubmed/?term=Joshi%20KK%5BAuthor%5D&cauthor=true&cauthor_uid=3278292). Meningococcal brain abscess and meningitis in a neonate. [Pediatr Infect Dis J.](https://www.ncbi.nlm.nih.gov/pubmed/3278292) 1988 Feb;7(2):136-7. | PMID: 3278292 |  | No | English |
| Basani L, Aepala R.  Neisseria Meningitidis Causing Multiple Cerebral Abscesses in Early Neonatal Period: Case Report and Review of Literature. J Clin Diagn Res. 2017;11(7):SD01-SD03. | PMID: 28892991      DOI: [10.7860/JCDR/2017/25284.10151](https://doi.org/10.7860/JCDR/2017/25284.10151) | Yes |  | English |
| Ray S, Riordan A, Tawil M, Mallucci C, Jauhari P, Solomon T, Kneen R.  Subdural Empyema Caused by Neisseria meningitidis: A Case Report and Review of the Literature. Pediatr Infect Dis J. 2016;35(10):1156-9. | PMID:27254039    DOI:[10.1097/INF.0000000000001252](https://doi.org/10.1097/INF.0000000000001252) | Yes |  | English |
| Rothbaum E, Nicholson O, Prince A. Cerebral abscess associated with meningococcal meningitis. Pediatr Infect Dis J. 2006;25(8):754-6 | PMID:16874183    DOI:[10.1097/01.inf.0000227808.81951.74](https://doi.org/10.1097/01.inf.0000227808.81951.74) | Yes |  | English |
| Faisant C, Cochin JP, Rapoport N, Evreux F, Vaschalde Y.  [Cerebral trunk abscess due to Neisseria meningitidis in a 28-year-old immunocompetent patient.](https://www.ncbi.nlm.nih.gov/pubmed/22284670) Rev Neurol (Paris). 2012 Mar;168(3):287-90. | doi: 10.1016/j.neurol.2011.08.014. |  | No | French |
| Jouhadi Z, Touki A, Dreoua N, Daoud N, Najib J, Abid A.  Sub-dural abscess and empyema: unusual complications of meningococcal meningitis: four paediatric cases. Med Mal Infect. 2004 Jun;34(6):270-2. | PMID:15612360 |  | No | French |
| Edwards MS, Baker CJ.  [Subdural empyema: an unusual complication of meningococcal meningitis.](https://www.ncbi.nlm.nih.gov/pubmed/7054884) South Med J. 1982 Jan;75(1):68-9. | PMID: 7054884 |  | No | English |
